# Cellular aging is accelerated in the malignant clone of myeloproliferative neoplasms

**DOI:** 10.1038/s41408-023-00936-1

**Published:** 2023-11-06

**Authors:** Margherita Vieri, Vithurithra Tharmapalan, Milena Kalmer, Julian Baumeister, Miloš Nikolić, Matthis Schnitker, Martin Kirschner, Niclas Flosdorf, Marcelo A. S. de Toledo, Martin Zenke, Steffen Koschmieder, Tim H. Brümmendorf, Fabian Beier, Wolfgang Wagner

**Affiliations:** 1 Department of Hematology, Oncology, Hemostaseology and Stem Cell Transplantation, Medical Faculty of RWTH Aachen University, University Hospital Aachen, 52074 Aachen, Germany; 2 Center for Integrated Oncology Aachen Bonn Cologne Düsseldorf (CIO ABCD), Aachen, Germany; 3 Helmholtz-Institute for Biomedical Engineering, Medical Faculty of RWTH Aachen University, 52074 Aachen, Germany; 4 Institute for Stem Cell Biology, Medical Faculty of RWTH Aachen University, 52074 Aachen, Germany; 5 Institute for Biomedical Engineering—Cell Biology, Medical Faculty of RWTH Aachen University, 52074 Aachen, Germany

**Keywords:** Myeloproliferative disease, Cancer epigenetics

Dear Editor,

Myeloproliferative neoplasms (MPNs) are caused by somatic driver mutations, such as *JAK2*^V617F^, which might also affect cellular aging and senescence. Here, we analyzed the heterogeneity of aging in MPN patients and if this can be used to specifically target malignant cells. Our results indicate that cellular aging is accelerated in malignant MPN clones and this can provide a target for treatment with senolytic drugs or telomerase inhibitors.

Aging is reflected by epigenetic modifications, such as changes in DNA methylation (DNAm). We utilized an epigenetic age–predictor based on amplicon deep-sequencing of three age-associated regions (Supplementary Methods) [1]. In healthy donors, there was a high correlation of predictions with chronological age (R^2^ = 0.86**)** with a mean age deviation (MAD) of 0.8 years (Supplementary Fig. S1a). In contrast, epigenetic age-predictions of 128 MPN patients showed much higher offsets, particularly in the advanced MPN entity primary myelofibrosis (PMF; Fig. 1a). Overall, epigenetic aging was rather increased, resulting in a positive delta-age (predicted age—chronological age; Fig. 1b).

**Fig. 1:**
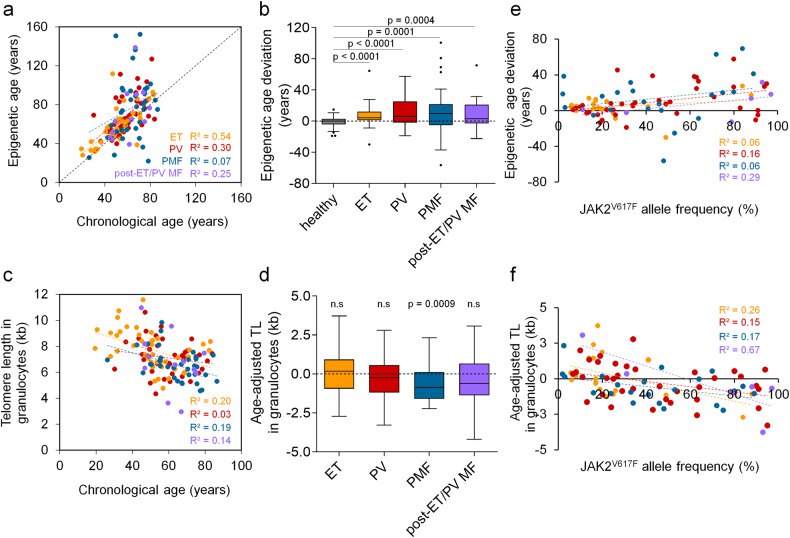
Cellular aging is progressively accelerated in MPN entities. **a** Correlation of chronological age and epigenetic age predictions by bisulfite barcoded amplicon sequencing (BA-seq) of three CpGs in MPN patients (*n* = 128; ET essential thrombocythemia, PV polycythemia vera, PMF primary myelofibrosis). **b** Epigenetic age deviation in different MPN entities compared to healthy donors. Unpaired *t*-test was used to assess statistical significance. **c** Telomere length (TL, in kb) was measured in granulocytes via flow-FISH in blood of MPN patients (*n* = 128). **d** Age-adapted TL in granulocytes in different MPN entities. One-sample t-test was used to calculate statistical significance. **e** Correlation of epigenetic age deviation and *JAK2*^V617F^ allele burden in different MPN entities. **f** Correlation of age-adjusted TL and *JAK2*^V617F^ allele burden.

Telomere length (TL) attrition represents another denominator of cellular aging and its impact has been studied extensively in chronic myeloid leukemia (CML), where TL is shortened in the malignant stem cell clone [2]. Flow-FISH analysis of TL in granulocytes revealed a clear association with chronological age in 134 healthy donors, albeit the correlation was lower than for epigenetic age-prediction (R^2^ = 0.18; Supplementary Fig. S1b). In the 128 MPN samples, the distribution was overall similar (Fig. 1c), while a significant TL attrition was only observed for the PMF samples (*p* = 0.0009; Fig. 1d). Interestingly, there was only a moderate correlation between TL attrition and epigenetic delta-age (Supplementary Fig. S1c). Thus, TL and epigenetic age seem to reflect different biological properties of aging.

Next, we analyzed if these measures for cellular aging were associated with specific somatic mutations using a next-generation sequencing panel of 32 genes. Epigenetic age deviation showed a significant increase in patients with *JAK2*^*V617F*^ (*p* < 0.001) and *CALR* mutations (*p* = 0.013; Supplementary Fig. S1d), and age-adjusted TL in granulocytes showed significantly accelerated shortening in samples with mutation in *CALR* (*p* = 0.025) and *ASXL1* (*p* = 0.004; Supplementary Fig. S1e). Notably, in patients carrying the *JAK2*^V617F^ mutation, there was a significant association of epigenetic age acceleration (*p* = 0.0031; Fig. 1e), similar to what was shown in polycythemia vera (PV) in another study [3], as well as telomere attrition (*p* < 0.0001; Fig. 1f) with mutational burden if all MPN subgroups were combined.

To further investigate cellular aging in the malignant clone of patients with MPN, we analyzed single cell-derived colony forming units (CFUs). Epigenetic age predictions were exemplarily performed for 5 wild type (WT) and 5 *JAK2*^V617F^ colonies for an individual patient per MPN sub-entity. There was some variation between individual colonies, but overall, the epigenetic age predictions were consistently higher for the mutated clones (Fig. 2a). In analogy, we analyzed TL in individual *JAK2*^V617F^ and *JAK2*^WT^ colonies with TEL-PCR in 10 PV patients and 7 healthy donors (Fig. 2b and Supplementary Fig. S2a). Overall, the mean TL of colonies with *JAK2*^V617F^ mutation was significantly shorter than in WT colonies (*p* = 0.0075). The degree of telomere shortening in *JAK2*^V617F^ colonies (ΔTL^mut-WT^) was found to correlate significantly with the patient’s allele burden (Supplementary Fig. S2b). However, these associations were not observed in 7 PMF patients (Fig. 2c and Supplementary Fig. S2c, d).

**Fig. 2:**
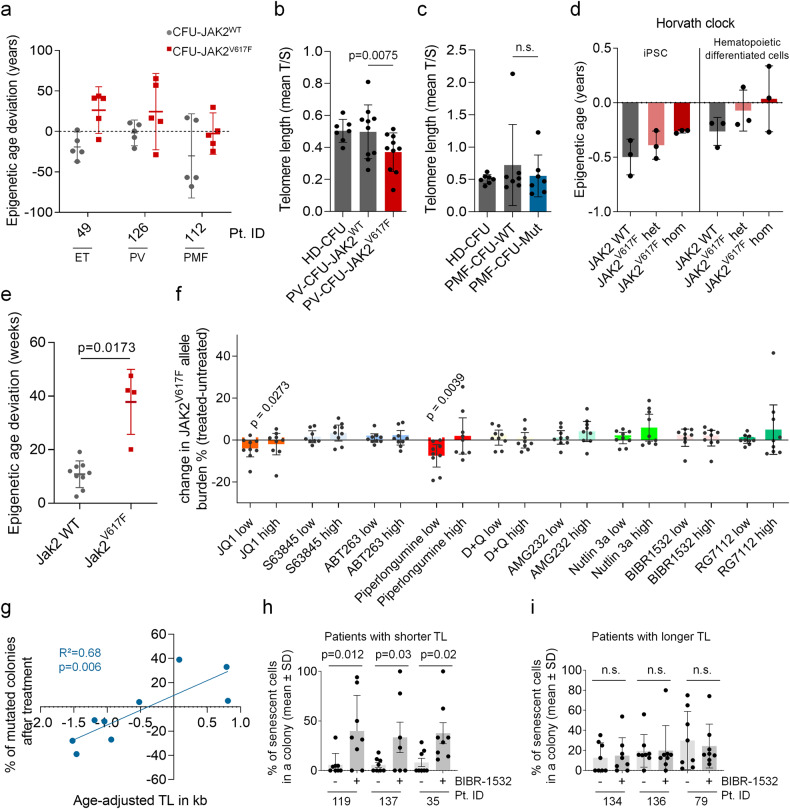
Cellular aging is accelerated in malignant clones, providing a rationale for treatment with senolytic drugs or telomerase inhibitors. **a** Epigenetic age predictions were performed in colony forming units (CFUs) without (*n* = 5) and with *JAK2*^V617F^ (*n* = 5) in three different patients affected by either ET, PV, or PMF. **b** Telomere length analysis (TEL-PCR) in single colonies derived from ten PV patients (all positive for JAK2V617F) and seven healthy donors (HD). For each patient, up to ten colonies were analyzed also for *JAK2*^V617F^ genotype and the mean difference in TL between WT and *JAK2*^V617F^ colonies of each individual PV patient was calculated (paired *t*-student test). **c** In analogy, TL was analyzed in up to ten single colonies derived from seven PMF patients and the same seven healthy donors. **d** Syngeneic iPSC lines with JAK2 WT, heterozygous, or homozygous JAK2^V617F^ mutation were differentiated towards hematopoietic lineage. Epigenetic age was estimated in iPSC and iPSC-derived hematopoietic cells base on Illumina BeadChip profiles using Horvath epigenetic clock epigenetic signature. **e** Vav-iCre transgenic (tg/+) mice were crossed with conditional knock out mice for Jak2^V617F^ (flox/+) in order to express the mutation in the hematopoietic lineage. Epigenetic age deviation in bone marrow derived cells from WT mice (gray) and Jak2^V617F^ mice (red) was then compared (unpaired Welch’s *t*-test). **f** Peripheral blood mononuclear cells of MPN patients were cultured for three days with nine different compounds at concentrations below and above the IC50 (indicated as low and high concentration): JQ1 (10 µM, 20 µM), S63845 (500 nM, 1 µM), ABT-263 (100 nM, 200 nM), Piperlongumine (10 µM, 50 µM), Dasatinib in combination with Quercetin (D + Q; 20 µM, 50 µM), AMG-232 (1 µM, 10 µM), Nutlin-3a (10 µM, 50 µM), BIBR-1532 (50 µM, 100 µM), and RG-7112 (10 µM, 50 µM). *JAK2*^V617F^ mutational burden was measured by ddPCR in untreated *versus* treated cells (*n* = 9; one-sample *t*-test). **g** Peripheral blood mononuclear cells of nine PMF patients were seeded into a colony forming unit assay for 14 days either with BIBR-1532 (50 µM) or DMSO for control. The change in *JAK2*^V617F^ or *CALR* mutational burden was measured before and after treatment and correlated with the age-adjusted TL (measured by flow-FISH). **h** Percentage of senescent cells measured after ß-galactosidase staining in eight colonies analyzed after treatment with BIBR-1532 (50 µM) or DMSO. Colonies were derived from patients with shorter telomeres (Patient# indicated). **i** The same analysis was performed for patients with longer telomeres (paired t-student test).

To understand if the *JAK2*^V617F^ mutation directly affects cellular aging parameters, we used previously established syngeneic induced pluripotent stem cell (iPSC) models [4]. Corresponding iPSC lines were generated from three MPN patients with WT *JAK2*, heterozygous and homozygous *JAK2*^V617F^ mutations. These clones were then differentiated toward the hematopoietic lineage (Supplementary Fig. S3a). Here, we focused particularly on the cellular aging in the iPSC-lines and iPSC-derived hematopoietic cells. To estimate epigenetic age, we used two multi-tissue epigenetic predictors: the Horvath epigenetic clock [5] (Fig. 2d) and Horvath Skin and Blood clock [6] (Supplementary Fig. S3b). There was a tendency towards increased epigenetic age in the *JAK2*^V617F^ mutation, but the predictions were overall close to 0 years, as generally observed upon reprogramming [7]. Furthermore, TL analysis did not reveal consistent telomere shortening in the *JAK2* mutant clones (Supplementary Fig. S3c). Thus, the impact of *JAK2*^V617F^ on acceleration of cellular aging appears to be minimal upon short-term differentiation in the iPSC model.

Next, we analyzed vav-cre driven *Jak2*^V617F^ transgenic mice after development of an MPN-like phenotype (Supplementary Fig. 4a) [8]. When we analyzed epigenetic age in unfractionated bone marrow cells, there was a significant acceleration in mice with *Jak2*^V617F^ mutation (*n* = 4) as compared to WT *Jak2* (*n* = 9; Fig. 2e, *p* = 0.017). TL did not differ between *Jak2*^WT^ and *Jak2*^V617F^ (Supplementary Fig. S4b), which might be due to different expression of telomerase and regulation of TL in mice.

Senolytic drugs raised hopes to specifically target prematurely aged cells that show signs of senescence and to thereby rejuvenate tissues [9]. We anticipated that particularly the malignant clones, revealing signs of accelerated aging and displaying a senescence-associated gene signature (Supplementary Fig. S5), might be more susceptible to senolytic drugs than the remaining non-mutated cells. We cultured peripheral blood mononuclear cells (PBMCs) of nine MPN patients with *JAK2*^V617F^ mutation for three days with eight different senolytic drugs using the concentrations below and above the IC50: piperlongumine, ABT-263, RG-7112, nutlin-3a, dasatinib in combination with quercetin, AMG-232, JQ1, and S63845. Furthermore, we tested the telomerase inhibitor BIBR-1532. After three days, the remaining *JAK2*^V617F^ mutation burden was analyzed with digital droplet PCR. Overall, the senolytic drugs had only a moderate specific effect on the mutated subsets. Only JQ1, a potent inhibitor of the BET family of bromodomain proteins, and piperlongumine, an amide alkaloid constituent of the long pepper, showed a moderate but significant reduction of the *JAK2*^V617F^ allele burden (for piperlongumine only at low concentration; Fig. 2f). Simultaneously, we analyzed if the treatment would also affect epigenetic age predictions (*n* = 7 for each compound). A moderate, non-significant reduction of epigenetic age-predictions was observed for RG7112 and again for piperlongumine (Supplementary Fig. S6a), potentially due to the depletion of clonal cells with premature epigenetic age. Furthermore, we measured TL with TEL-PCR (*n* = 5 for each compound). A significant increase in TL was again observed with JQ1, piperlongumine and nutlin-3a (Supplementary Fig. S6b). Notably, Kleppe and colleagues demonstrated a reduction in disease burden of MPN by JQ1 treatment either alone or in combination with ruxolitinib [10]. Taken together, at least JQ1 and piperlongumine might have a selective effect for malignant cells with accelerated cellular aging, although the compounds may also have non-senolytic effects, particularly at these relatively high concentrations during short-term in vitro treatment.

The use of telomerase inhibitors (TI) is an option to target malignant cells with shorter telomere length. BIBR-1532 is a potent telomerase inhibitor that has been tested previously in CML [11], but not yet systematically on BCR-ABL-negative MPN cells. Although short-term treatment with BIBR-1532 did not reduce the prominent malignant clone with accelerated aging, we hypothesize that inhibition of telomerase mediates proliferation-dependent critical telomere shortening, which eventually leads to telomere-mediated senescence or apoptosis. Therefore, an effect on subclones with particularly short TL might be observed only after longer treatment. To this end, we cultured PMF-derived PBMC (*n* = 9) in CFU assays with or without BIBR-1532, picked 30 colonies per condition, and analyzed the percentage of mutated colonies (*JAK2*^V617F^ and *CALR* rearrangements). Notably, the fraction of mutated colonies declined particularly in those patients with preexisting shorter mean TL in granulocytes by flow-FISH analysis (R^2^ = 0.68; *p* = 0.0064; Fig. 2g), indicating that samples with accelerated telomere attrition are more susceptible to telomerase inhibition.

Subsequently, we performed ß-galactosidase staining within individual colonies (Supplementary Fig. S7a). In the three PMF donors with very short telomeres, the fraction of senescent cells clearly increased upon BIBR-1532 treatment (Fig. 2h). In contrast, this effect was not observed in the three patients that revealed longer telomeres (Fig. 2i). To further substantiate if the potential triggering of senescence occurs specifically in the malignant cells, we further analyzed the genotype in these colonies. BIBR-1532 treatment seems to specifically increase the fractions of senescent cells in CFUs carrying *JAK2*^V617F^ mutation or CALR rearrangements in patients with shorter telomeres (Supplementary Fig. S7b, c). While BIBR-1532 has so far not been evaluated in clinical trials, telomerase inhibition with imetelstat has already demonstrated clinical activity in primary, post-essential thrombocythemia or post-PV myelofibrosis [12–14]. Another study on non-small cell lung cancer cell lines demonstrated that particularly those lines with short TL were susceptible to imetelstat treatment [15]. It will, therefore, be important to determine if particularly patients with short telomeres at diagnosis profit from imetelstat treatment and if this telomerase inhibitor might also induce senescence in the mutant compartment of MPN. It might even be conceivable to combine TI and senolytics to accelerate senescence specifically in the malignant stem cell clones in MPN patients (by the TI component) and to finally eradicate it (with a senolytic drug).

### Supplementary information

Supplementary methods, figures, and tables

Supplementary Table S1

## Data Availability

The datasets used and/or analyzed in this current study are available from the corresponding author on reasonable request.
