# Supplementary material for: Cellular aging is accelerated in the malignant clone of myeloproliferative neoplasms
Source: Blood Cancer J. 2023 Nov 6;13(1):164. doi: 10.1038/s41408-023-00936-1 (PMC10625927; doi:10.1038/s41408-023-00936-1)
Supplement: Supplementary file 1 — Supplementary methods, figures, and tables [file 41408_2023_936_MOESM1_ESM.pdf]

Supplemental Information

Cellular aging is accelerated in the malignant clone of myeloproliferative neoplasms

Margherita Vieri[1,2,*], Vithurithra Tharmapalan[2,3,4,*], Milena Kalmer[1,2], Julian Baumeister[1,2], Miloš Nikolić[2,3,4], Matthis Schnitker[2,3,4], Martin Kirschner[1,2], Niclas Flosdorf[3,5], Marcelo A. S. de Toledo[1,2], Martin Zenke[1,2,3,5], Steffen Koschmieder[1,2], Tim H. Brümmendorf[1,2,*], Fabian Beier[1,2,*], and Wolfgang Wagner[2,3,4,*]

[1] Department of Hematology, Oncology, Hemostaseology and Stem Cell Transplantation, Medical Faculty of RWTH Aachen University, University Hospital Aachen, 52074 Aachen, Germany

[2] Center for Integrated Oncology Aachen Bonn Cologne Düsseldorf (CIO ABCD)

[3] Helmholtz-Institute for Biomedical Engineering, Medical Faculty of RWTH Aachen University, 52074 Aachen, Germany

[4] Institute for Stem Cell Biology, Medical Faculty of RWTH Aachen University, 52074 Aachen, Germany

[5] Institute for Biomedical Engineering – Cell Biology, Medical Faculty of RWTH Aachen University, 52074 Aachen, Germany

* These authors contributed equally to this work

Corresponding author: Wolfgang Wagner, Helmholtz-Institute for Biomedical Engineering, Institute for Stem Cell Biology, Medical Faculty of RWTH Aachen University, Pauwelsstrasse 20, Aachen, Germany, Phone: +49 241 8088611; E-mail: wwagner@ukaachen.de

Supplemental Methods 2
- Blood samples 2
- Analysis of mutational burden 2
- Telomere length measurements 2
- Epigenetic age prediction with targeted bisulfite amplicon sequencing 2
- Epigenetic age prediction with pyrosequencing 3
- Colony forming unit assay 3
- Analysis of genes associated with senescence in the microarray data 3
- Senescence associated beta-galactosidase (SA-ß-gal) assay 3
- Generation and hematopoietic differentiation of *JAK2*^{V617F} iPSC 3
- DNA methylation analysis using BeadChip data 4
- Jak2^{V617F} mouse model 4
- Testing of senolytic drugs and a telomerase inhibitor 4
- Statistics 5

Supplemental Figures 6
- Supplementary Figure S1. Cellular aging is progressively accelerated in MPN entities with specific mutations 6
- Supplementary Figure S2. Heterogeneity of TL length in colony forming units 7
- Supplementary Figure S3. iPSC model to investigate the effect of JAK2V617F mutation on cellular aging. 8
- Supplementary Figure S4. Murine model to investigate the effect of JAK2V617F mutation on cellular aging. 8
- Supplementary Figure S5: Senescence phenotype is increased in MPN. 9
- Supplementary Figure S6. Testing the effect of senolytic compounds on parameters of cellular aging 10
- Supplementary Figure S7. Telomerase inhibitor evokes senescence in mutated colonies with short telomeres 11

Supplemental Tables 11
- Table S1: Overview of samples and clinical data of MPN patients 11
- Table S2: PCR conditions for bisulfite barcoded amplicon sequencing 11
- Table S3: Primer list for the genotyping of single CFU colonies 12

Supplemental References 12

Supplemental Methods

Blood samples

Peripheral blood samples of 153 patients diagnosed with MPN were used. A detailed description of the patient cohort is shown in Supplemental Table S1. Blood samples of 134 healthy donors were used for age-adaptation of TL measured via flow-FISH (1). Furthermore, a separate cohort of 128 healthy controls was used for the measurement of epigenetic age deviation (2). All samples were obtained after informed and written consent in accordance with the Declaration of Helsinki and the research was specifically approved by the local ethics committee of RWTH Aachen University (EK 041/15, EK 206/09 and EK 127/12).

Analysis of mutational burden

A clinically validated amplicon-based next-generation sequencing (NGS) panel (Truseq Custom Amplicon Kit, Illumina, San Diego, USA) was used to analyze the coding region of 32 genes that typically harbor mutations associated with hematologic malignancies, as described previously (3, 4). Variants were reviewed manually, using a bidirectional frequency of >1% for driver mutations and > 5% for additional mutations as cutoffs. Alternatively, the $JAK2^{V617F}$ allele burden was analyzed with digital droplet PCR (ddPCR) using the mutation assay dHsaMDV27944642 from Bio-Rad, according to the manufacturer's instructions.

Telomere length measurements

Flow-fluorescent *in situ* hybridization (flow-FISH) (1, 5): Cryopreserved whole blood cells after red blood cell depletion were used for the flow-FISH analysis of telomere length (TL) in granulocytes, as previously described (1). Briefly, samples were prepared for cell denaturation and mixed with a telomere-specific (CCCTAA)3-peptide nucleic acid FISH probe labeled with FITC (Eurogentec, Liège, Belgium) for DNA hybridization. DNA counterstaining was performed with LDS 751 (Sigma-Aldrich, Missouri, USA). Granulocytes are stained by LDS 751 and can be distinguished by signal intensities in both the FL-3 channel as well as in the forward scatter (FSC). TL of bovine thymocytes was determined by Western blot (19.515 kb) and used as a reference to convert the TL of granulocytes into kb. An FC 500 flow cytometer (Becton Dickinson, East Rutherford, USA) was used for data acquisition. All measurements were carried out in triplicates. Healthy controls (n = 134) were used for age adaptation of TL.

Telomere PCR (TEL-PCR) (6): This method was used for TL measurement in colony forming units (CFUs), iPSCs before and after hematopoietic differentiation and in mouse bone marrow-derived cells. 1.4 ng of genomic DNA per reaction was used in the Absolute Human TL Quantification qPCR Assay Kit (ScienCell, Carlsbad, USA) and FastStart Essential DNA Green Master (Roche, Basel, Switzerland). TL measurements are given in T/S ratios. A T/S ratio is calculated by dividing the number of copies of the telomere template (T) by the single copy reference (SCR) template (S), which is an amplified 100 bp region on human chromosome 17. The TL q-RT-PCR was performed according to the manufacturer's instructions.

Epigenetic age prediction with targeted bisulfite amplicon sequencing

Genomic DNA was isolated from cryopreserved whole blood cells after red blood cells depletion with the QIAamp DNA Mini Kit (Qiagen, Hilden, Germany), or for colonies in the CFU assay with NucleoSpin XS Tissue Kit (Macherey-Nagel, Düren, Germany). DNA was quantified with a Nanodrop 2000 Spectrophotometer (Thermo Scientific, Wilmington, USA) and bisulfite converted with the EZ DNA Methylation Kit (Zymo Research, Irvine, USA). For targeted bisulfite amplicon sequencing (BA-seq) three age-associated CG dinucleotides (CpG sites) that are associated with the coiled-coil domain-containing protein 102B (*CCDC102B*), four and a half LIM domains protein 2 (*FHL2*), and phosphodiesterase 4C (*PDE4C*) (2) were amplified by PyroMark PCR kit (Qiagen) using primers with handle sequences for the subsequent barcoding step, as described in detail before (2). PCR conditions are summarized in Supplemental Table S2. The three amplicons of each donor were pooled at equal concentrations, quantified with Qubit (Invitrogen, Massachusetts, USA), and cleaned up with paramagnetic beads from Agencourt

AMPure PCR Purification system (Beckman Coulter, California, USA). Four microliters of PCR products were subsequently added to 21 µl PyroMark Master Mix (Qiagen) containing 0.4 µM of barcoded primers (adapted from NEXTflexTM 16S V1-V3 Amplicon Seq Kit, Bioo Scientific, Austin, USA) for a second PCR. PCR products were again quantified with the Qubit, combined in equimolar ratios, and cleaned by Select-a-Size DNA Clean & Concentrator Kit (Zymo Research). A 12-pM DNA library was diluted with 15% PhiX spike-in control and eventually subjected to 250 bp pair-end sequencing on a MiSeq lane using the Miseq reagent V2 Nanokit (both from Illumina).

FastQ files from MiSeq analysis were aligned to the reference genome *hg19* using the Bismark tool (7) and DNA methylation values determined with the Bismark methylation extractor. Epigenetic age was calculated as follows:

Predicted age (in years) = $3.86 + 0.825\ \text{DNAm}^{FHL2} - 0.342\ \text{DNAm}^{CCDC102B} + 1.177\ \text{DNAm}^{PDE4C}$

Epigenetic age prediction with pyrosequencing

Pyrosequencing was used for fast and robust epigenetic age predictions of mouse bone marrow cell pellets and peripheral blood mononuclear cells (PBMCs) from patients after senolytic drug treatment *in vitro*. For mouse epigenetic age, three age-associated CpG sites were measured in Proline rich membrane anchor 1 (*Prima1*), Heat shock transcription factor 4 (*Hsf4*) and Potassium voltage-gated channel modifier subfamily S member 1 (*Kcns1*), as described in our previous work (8) and updated by selecting neighboring CpGs for the Prima1 region. Epigenetic age was calculated as follows:

Predicted age (in weeks) = $-6.325 - 0.308\ \text{DNAm}^{Prima1} + 2.588\ \text{DNAm}^{Hsf4} + 1.003\ \text{DNAm}^{Kcns1}$

For pyrosequencing analysis of the samples that were treated with senolytic compounds we analyzed for DNAm changes at the same age-associated CpGs of *PDE4C*, *CCDC102B* and *FHL2*, as described in detail before (2).

Colony forming unit assay

Peripheral blood mononuclear cells (PBMCs) of MPN and seven healthy donors were isolated by gradient centrifugation with Pancoll (Pan Biotech, Aidenbach, Germany) and $1\text{x}10^6$ cells per condition were transferred on a semisolid medium to perform CFU (9). After 14 days, colonies were counted and classified. To determine the mutation status of *JAK2* and *CALR* in single colonies, PCRs were performed for *JAK2*$^{\text{V617F}}$, *CALR*$^{\text{ins5}}$ and *CALR*$^{\text{del52}}$ (Supplemental Table S3) (9).

Analysis of genes associated with senescence in the microarray data

Gene expression profiles from 6 healthy donors, 6 ET, 11 PV and 9 PMF patients were published by Baumeister, J *et al.* in GSE174060 (10) and used for senescence pathway analysis (11). Significantly differentially regulated genes were selected by a Benjamini–Hochberg adjusted p value <0.05 and log2-fold changes above 0.5 or below −0.5. Enrichment of senescence-associated genes was estimated with hyper geometric distribution analysis.

Senescence associated beta-galactosidase (SA-ß-gal) assay

As a surrogate marker for senescence, we stained CFUs for β-galactosidase (Cell Signaling Technology, Danvers, USA). Single colonies were seeded on individual poly-L-lysine coated glass slide (Merck KGaA, Darmstadt, Germany) and stained according to the manufacturer's instructions. Cells were observed under a microscope (Leica DMRX, Leica Microsystems, Wetzlar, Germany).

Generation and hematopoietic differentiation of *JAK2*$^{\text{V617F}}$ iPSC

Induced pluripotent stem cells (iPSC) from three PV patients were generated by reprogramming PBMCs as described before (12, 13) (Flosdorf *et al.*, manuscript submitted). In patient 1 with 37% *JAK2*$^{\text{V617F}}$ allele burden in the PBMCs, only WT (Human Pluripotent Stem Cell Registry; UKAi002-A) and heterozygous (UKAi002-B) iPSC clones were obtained after reprogramming. Therefore, CRISPR/Cas9 genome engineering was used to introduce the JAK2$^{\text{V617F}}$ mutation generating a homozygous JAK2$^{\text{V617F}}$ iPSC clone (UKAi002-B3). Similarly, in patient 2 with 96% JAK2$^{\text{V617F}}$ allele burden in the PBMCs gave rise to only homozygous iPSC clones (UKAi003-A), and the

heterozygous (UKAi003-A2) and wild type ones (UKAi003-A1) were generated by CRISPR/Cas9 repair. In patient 3 with 25% JAK2^{V617F} allele burden in the PBMCs, only WT (UKAi016-A) and heterozygous (UKAi016-B) clones were obtained, and CRISPR/Cas9 was used to generate homozygous clones (UKAi016-B1).

Hematopoietic differentiation of iPSC clones was performed as described previously (14). In brief, iPSCs were cultured in micro-contact printed plates with StemMACS iPS Brew XF medium (Miltenyi Biotec) and 10 µM Y-27632 (Abcam, Cambridge, United Kingdom) to form embryonic bodies (EB) (15). Self-detachment of EBs was observed after 6 to 9 days, depending on the clone, and they were then resuspended in serum-free medium containing 50% IMDM, 50% Ham's F12, 1% chemically defined lipid concentrate, 2 mM GlutaMAX (all Thermo Fisher Scientific), 0.5% Albiomin (Unifols), 400 µM 1-thioglycerol, 50 µg/mL L-ascorbic acid, and 6 µg/mL holo transferrin (all Sigma Aldrich, St. Louis, MO, USA) supplemented with 10 ng/mL FGF-2 (Peprotech, Hamburg, Germany) and 10 ng/mL BMP-4 (Miltenyi Biotec). Approximately 30 to 50 EBs were distributed per well on a gelatin coated 6-well plate. From day 2 to day 7, cells were cultured in serum-free medium supplemented with 10 ng/mL FGF 2, 10 ng/mL BMP-4, 50 ng/mL SCF, 10 ng/mL VEGF-A (all Peprotech), and 10 U/mL penicillin/streptomycin (Thermo Fisher Scientific). From day 8 to day 16, serum-free medium was supplemented with 10 ng/mL FGF 2 and 50 ng/mL SCF only. Cells were harvested on day 16 and their phenotype was analyzed by flow cytometry and colony forming assay. For details on the hematopoietic differentiation and characterization we refer to our previous work (14).

DNA methylation analysis using BeadChip data

For iPSC and iPSC-derived hematopoietic cells the above-mentioned targeted signatures for epigenetic age-prediction could not be applied, because they were specifically trained for primary hematopoietic cells. Thus, we used multi-tissue epigenetic age predictors for Illumina BeadChip data. Genomic DNA was isolated from the 9 iPSC clones and iPSC-derived hematopoietic differentiated cells with the QIAamp DNA Mini Kit (Qiagen, Hilden, Germany), and bisulfite converted and hybridized with the Illumina human EPIC methylation microarray at Life and Brain (Life and Brain GmbH, Bonn, Germany). Data was processed using the Sesame package for R (16), normalized and corrected for dye bias, background, and mask probes with poor design. Additionally, CpGs at the X and Y chromosomes were removed and also the probes with failed detection P values, with resulting in 630,700 CpGs. Epigenetic age was predicted using Horvath's clock (17) and Skin and blood Horvath clock (18) using watermelon package for R (19).

Jak2^{V617F} mouse model

The Vav-Cre-lox system was used to induce the *Jak2*^{V617F} mutation in the mouse model, as described previously (20). Six wild type (WT) and three heterozygous *Jak2*^{V617F} (VF) mice at 20 weeks of age and 3 WT and 1 VF mice at 30 weeks of age were used for epigenetic age prediction and telomere length measurements. The genotype of the mouse was confirmed by PCR, and massive splenomegaly and elevated myeloid and erythroid markers were observed in VF mice compared with WT mice. Bone marrow cells were collected for analysis by flushing tibiae and femurs, and red blood cells were lysed.

Testing of senolytic drugs and a telomerase inhibitor

Our choice of eight different senolytics was based on the different apoptotic mechanisms and the involvement of anti-apoptotic proteins, which have been shown to induce apoptosis in senescence cells. JQ1 is a BET inhibitor that targets non-homologous end joining to eliminate senescent cells, which is relevant in repairing the double stand breaks in DNA and activating the autophagy pathway (21). BH3-only BCL-2 family proteins are effectors of canonical mitochondrial apoptosis that have pro-apoptotic functions through BH1–3 pro-apoptotic proteins, such as BAX and BAK, while their activity is suppressed by BH1–4 anti-apoptotic BCL-2 family members (22). Here we used the first clinically approved BH3-mimetic ABT263 (Navitoclax) that target multiple BCL-2 proteins such as BCL-2, BCL-xL and BCL-w (23), and S63845 that selectively targets the anti-apoptotic protein MCL-1 (24). Dasatinib is a tyrosine kinase inhibitor, which interferes with EFNB- dependent suppression of apoptosis. Quercetin

has been shown to interfere with several anti-apoptotic pathways (25). Combination treatment showed a reduction in p16 and p21 expressing cells and SA-ß-gal positive cells (26). MDM2 inhibitors, which target the interaction of MDM2 with p53 and reactivate functional p53, lead the cell death of senescent cells (27). Depending on the binding site of p53-MDM2, different structurally unique small inhibitors were selected, such as AMG232, which is used in clinical trials in MPN patients who failed JAK inhibitor treatment (NCT03662126), RG7112, which is also used in clinical trials of leukemia patients (NCT01970930), and Nutlin-3a, which increases the degree of apoptosis in MPN by increasing p53 and p21 protein levels (28). Piperlongumine is a natural product that selectively kills cancer cells by inhibiting oxidative stress response proteins which are important for cancer cell survival in the presence of elevated ROS levels (29).

To estimate the senolytic activity of these compounds on cellular subsets with $JAK2^{V617F}$ mutation, we cultured cells for three days at different concentrations and analyzed proliferation and viability. PBMCs of MPN patients were cultured in StemSpan Serum-Free Expansion Medium (Stemcell Technologies, Vancouver, Canada), supplemented with 10 ng/mL SCF, 20 ng/mL TPO, 10 ng/mL FGF-1 (all PeproTech, Hamburg, Germany), 10 µg/mL heparin (Ratiopharm, Ulm, Germany), and 100 U/mL penicillin/streptomycin (Lonza, Basel, Switzerland) for three days. Selected nine drugs (eight senolytic compounds and the telomerase inhibitor BIBR-1532) were tested for their impacts on cell viability after three days with Cell Titer-Glo 2.0 luminescent cell viability assay (Promega, Wisconsin, USA) in 96-well plates that were seeded with 10 000 cells/well (3 wells per condition), using BioTek Synergy 2 plate reader and Gen5 software (Agilent Technologies, California, USA).

For a comparative approach of these compounds, we estimated the half-maximal inhibitory concentration (IC50) in our culture setting and used one concentration above and one below IC50 (diluted water or DMSO, according to the manufacturer's instructions): nutlin3a (10µM and 50µM (30), Selleck Chemicals LLC, Munich, Germany), JQ1 (10µM and 20µM (31), Sigma-Aldrich), ABT263 (100nM and 200nM (32), Selleck Chem), piperlongumine (10µM and 50µM (33), Selleck Chem), S63845 (500nM and 1µM (34), Selleck Chem), RG7112 (10µM and 50µM (35), Selleck Chem), dasatinib combined with quercetin (both 20µM and 50µM(36)), AMG232 (1µM and 10µM (37), Axon medchem, Groningen, Netherlands), and the telomerase inhibitor BIBR-1532 (50µM and 100µM (38), Selleck Chem). Alternatively, cells were cultured in a 24-well plate seeded with 250 000 cells/well (2 wells per condition) for DNA isolation. The effect of BIBR-1532 on the clonogenic potential was tested at a concentration of 50 µM in the CFU medium during 14 days of culture.

While the drug concentrations used in this study may appear relatively high, particularly with regard to long-term treatment or *in vivo* applications, they were at a similar range as described by other studies (Nutlin 3a, 10 µM (30); JQ1, 1-10 µM (31); ABT-263, <1µM (32); Piperlongumine, 10 µM (33); S63845, 5 nM -1 µM (34); RG 7112, 2.5- 5 µM (35); Dasatinib (D) + Quercetin (Q), 20 µM D + 15 µM Q, (39); AMG-232, 1 µM (37); and BIBR 1532, 50 µM (38)). However, at these concentrations some of the compounds may have additional effects beyond senolytics.

Statistics

Linear regressions, mean absolute deviation (MAD), and mean absolute error (MAE) of age-predictions were calculated with Excel. Statistical analysis was performed with GraphPad Prism using one sample t-test, unpaired t-test paired t-test or one-way ANOVA. P values ≤0.05 were considered as indicative of statistical significance. IC50 values were calculated by nonlinear regression analysis using GraphPad Prism.

Supplemental Figures

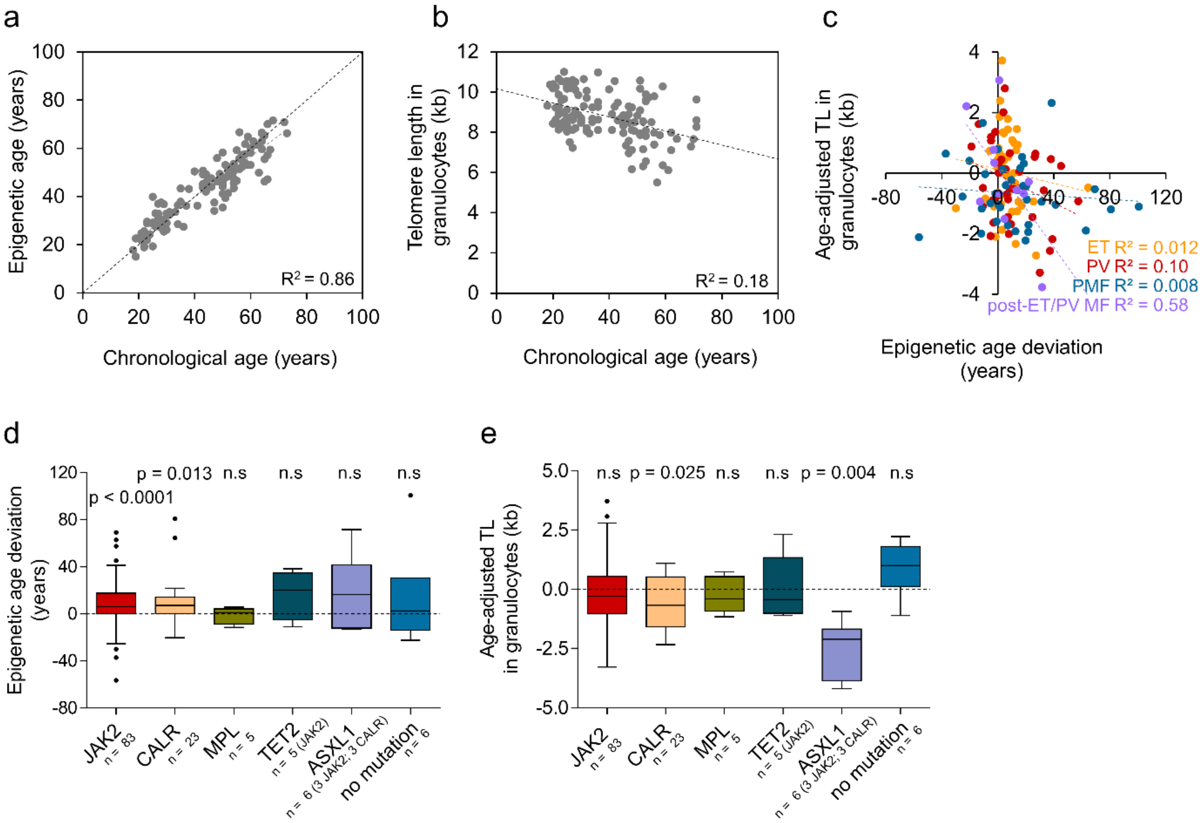

Supplementary Figure S1. Cellular aging is progressively accelerated in MPN entities with specific mutations

a) Correlation of chronological age and epigenetic age predictions by bisulfite barcoded amplicon sequencing (BA-seq) of three CpGs in healthy donors (n = 128). **b)** Telomere length (TL, in kb) was measured in granulocytes via flow-FISH in healthy donors, as described before (n = 134). **c)** Correlation of epigenetic age deviation with age-adjusted TL in granulocytes in MPN entities. **d)** Epigenetic age deviation in MPN carrying different driver mutations. Unpaired Welch's t-test was used to assess statistical significance. **e)** Age-adapted TL in granulocytes in MPN carrying different driver mutations. One-sample t-test was used to calculate statistical significance.

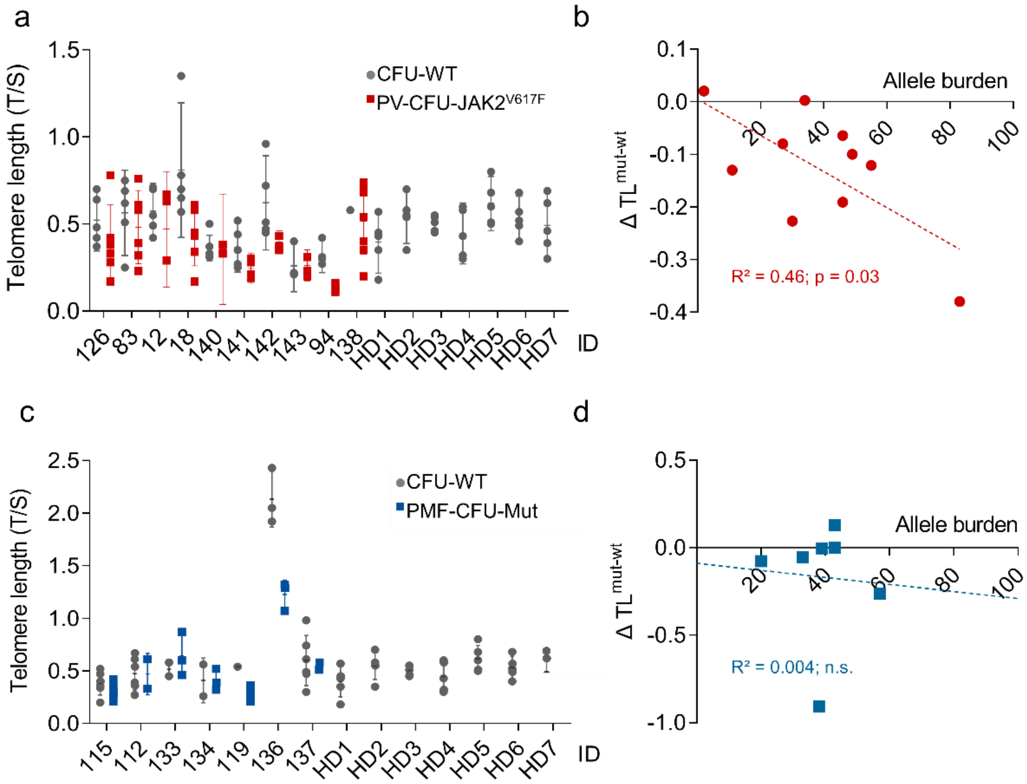

Supplementary Figure S2. Heterogeneity of TL length in colony forming units

a) Telomere length analysis (TEL-PCR) in single colonies derived from ten PV patients and seven healthy donors (HD). For each patient, up to ten colonies were analyzed also for *JAK2*^{V617F} genotype. **b)** Correlation between mean difference in TL between WT and *JAK2*^{V617F} with the initial *JAK2*^{V617F} allele burden of the patients. **c)** In analogy, TL was analyzed in up to then single colonies derived from seven PMF patients and the same seven healthy donors In the PMF patients. **d)** Correlation between the initial *JAK2*^{V617F} allele burden and the discrepancy in TL between mutated and non-mutated colonies.

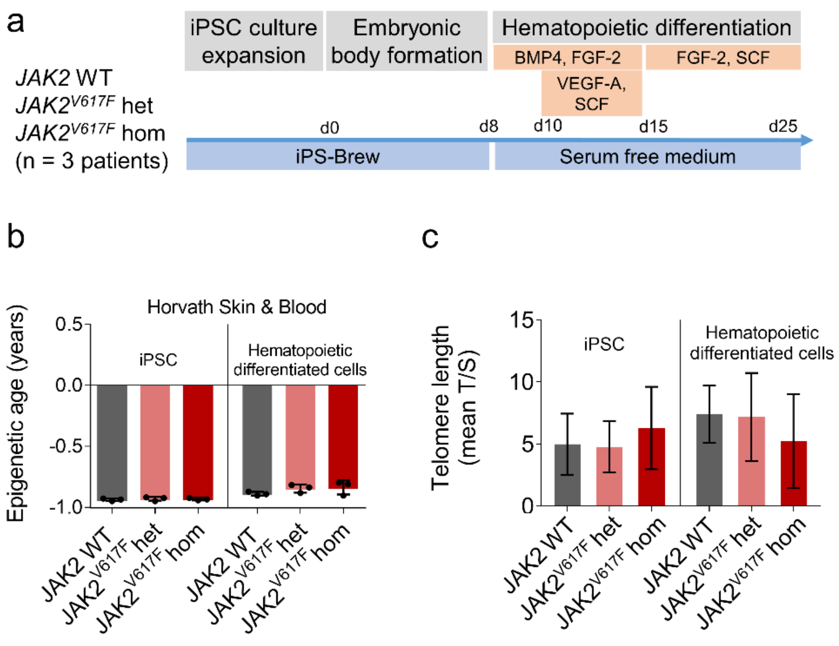

Supplementary Figure S3. iPSC model to investigate the effect of JAK2V617F mutation on cellular aging. **a)** Syngeneic iPSC lines with JAK2 WT, heterozygous, or homozygous JAK2V617F mutation were differentiated towards hematopoietic lineage. The differentiation protocol is indicated. **b)** Epigenetic age was estimated in iPSC and iPSC-derived hematopoietic cells base on Illumina BeadChip profiles using Horvath Skin and Blood clock. **c)** TL measurement of iPSCs and iPSC-derived hematopoietic cells according to their genotype.

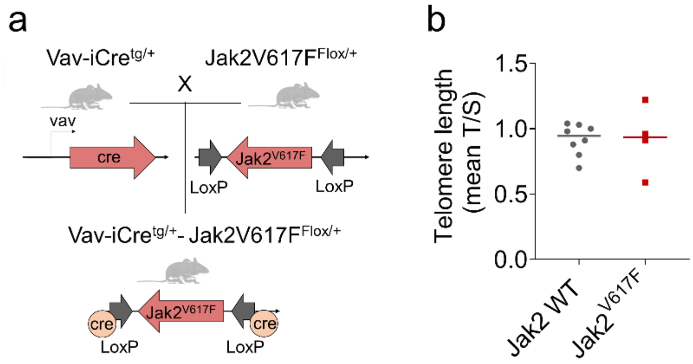

Supplementary Figure S4. Murine model to investigate the effect of JAK2V617F mutation on cellular aging. **A)** Schematic representation of the Jak2V617F mouse model system. Vav-iCre transgenic (tg/+) mice were crossed with conditional knock out mice for Jak2V617F (flox/+) in the hematopoietic lineage. **b)** Telomere length of WT *versus* Jak2V617F mice.

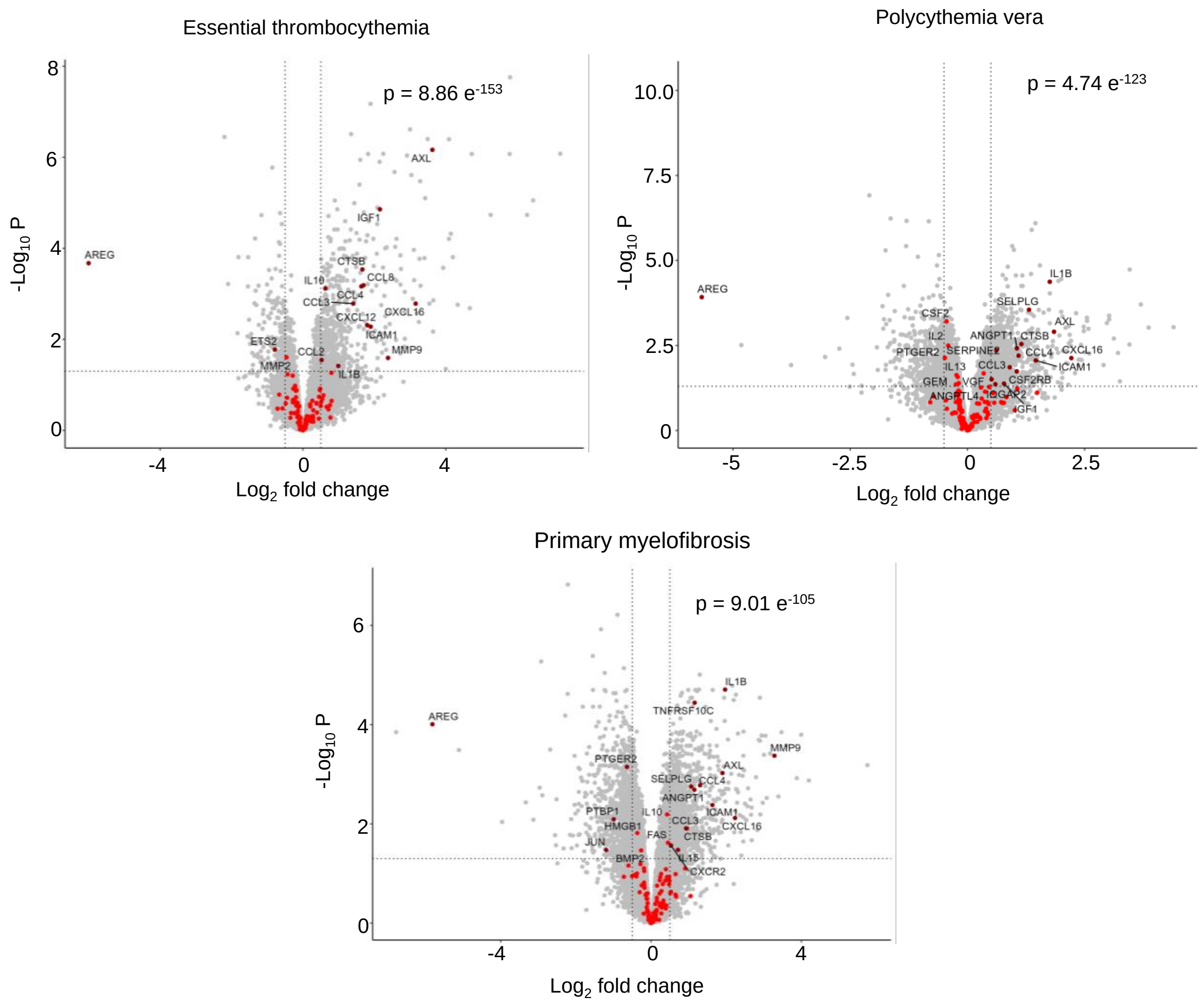

Supplementary Figure S5: Senescence phenotype is increased in MPN.

Gene expression profiles of 6 ET, 11 PV and 9 PMF patients, each compared with 6 healthy donors (GEO submission number GSE174060) and were analyzed for senescence-associated gene expression signatures. To this end, we focused on a set of 125 genes (SenMayo, indicated in red) that are differentially expressed during senescence. Significantly differentially regulated genes were selected by a Benjamini–Hochberg adjusted p value <0.05 and log2-fold changes above 0.5 or below −0.5. In fact, differential gene expression in MPN samples *versus* healthy donors revealed significant enrichment of this senescence-associated gene set for all MPN entities (p-value estimated by hyper geometric distribution)

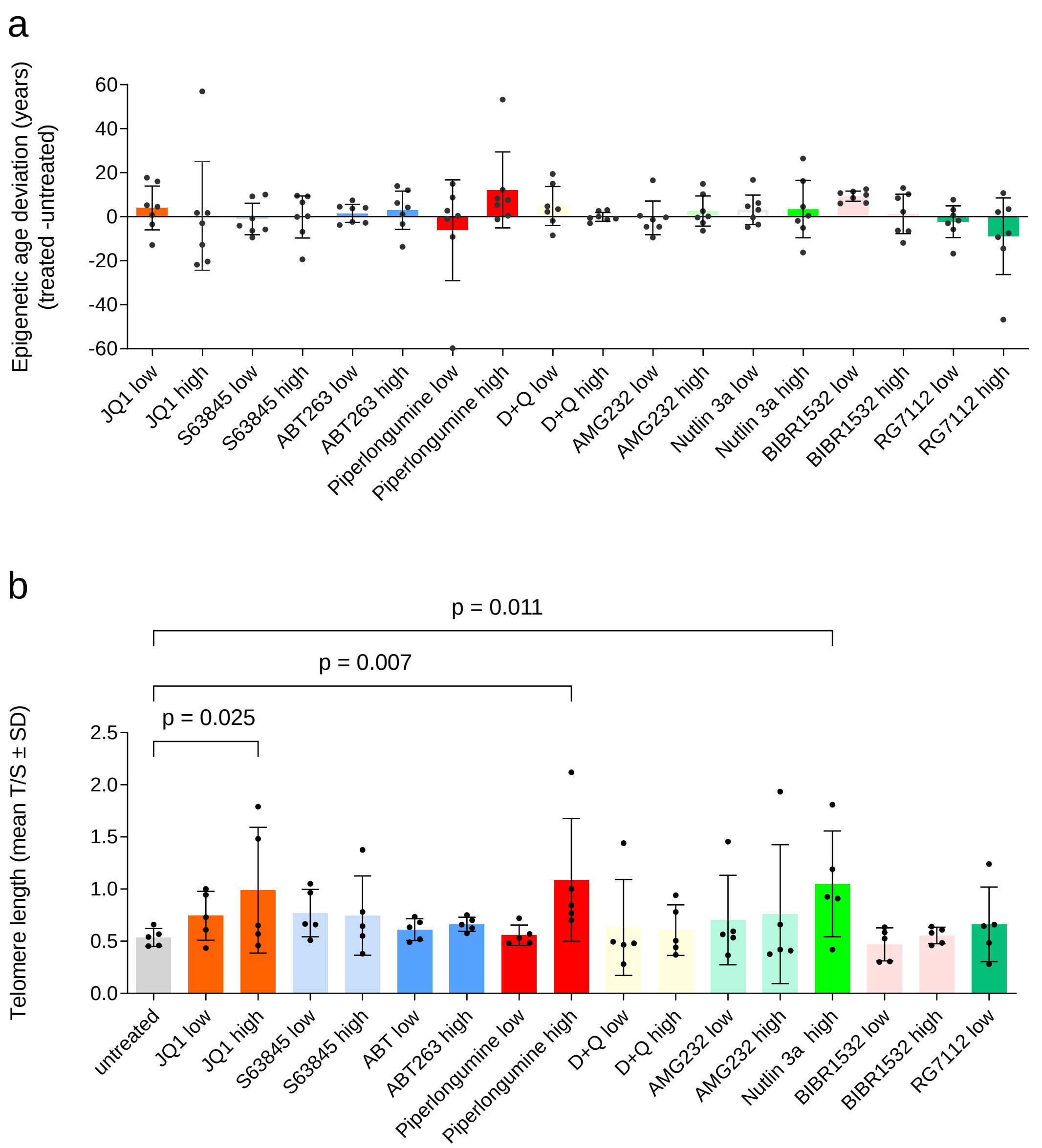

Supplementary Figure S6. Testing the effect of senolytic compounds on parameters of cellular aging

a) Epigenetic age changes in treated *versus* untreated cells (n = 7; measured by pyrosequencing; one-sample t-test). **b)** Changes in telomere length in treated *versus* untreated cells (n = 5; measured by TEL-PCR; one-way ANOVA). TL in Nutlin-3a 10 µM and RG7112 50 µM was not measurable due to a low concentration of DNA in the samples.

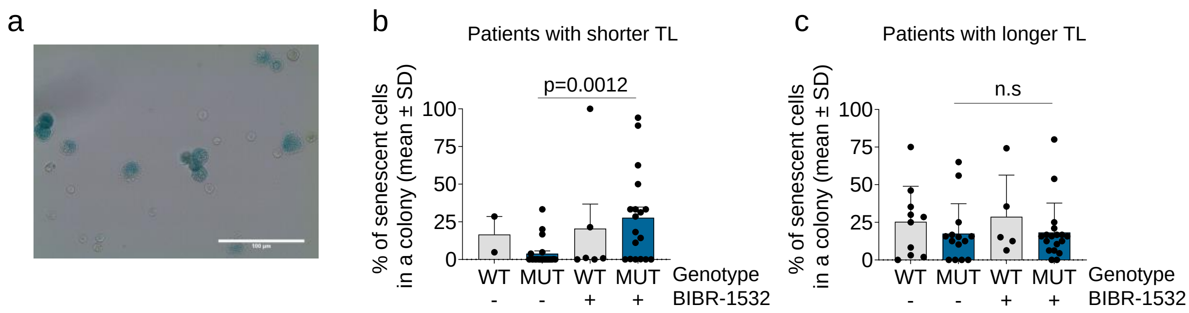

Supplementary Figure S7. Telomerase inhibitor evokes senescence in mutated colonies with short telomeres

a) ß-galactosidase staining within individual colonies depict senescent subsets. **b)** Percentage of senescent cells in WT and *JAK2*^{V617F} mutated or *CALR* rearranged colonies after treatment with BIBR-1532 (50 µM) or DMSO in the three patients analyzed in Figure 2h. **c)** This analysis is also shown for the three patients of Figure 2i (one-way ANOVA).

Supplemental Tables

Table S1: Overview of samples and clinical data of MPN patients

This table is provided as separate Excel file.

Table S2: PCR conditions for bisulfite barcoded amplicon sequencing

Step	PCR1 (*CCDC102B* and *FHL2*)			PCR1 (*PDE4C*)			PCR2		
	Temp.	Time	Cycles	Temp.	Time	Cycles	Temp.	Time	Cycles
Enzyme activation	95 °C	15 min		95 °C	15 min		95 °C	15 min	
Denaturation	95 °C	30 sec		95 °C	3 sec		95 °C	30 sec	
Annealing	56 °C	30 sec	40x	53 °C	35 sec	35x	60 °C	30 sec	16x
Extension	72 °C	30 sec		72 °C	35 sec		72 °C	30 sec	
Final extension	72 °C	10 min		72 °C	10 min		72 °C	10 min	
Hold	4 °C	∞		4 °C	∞		4 °C	∞	

1.5mM of $MgCl_2$ was used in both PCRs. These same conditions are used for all AML-associated regions.

Table S3: Primer list for the genotyping of single CFU colonies

Primer	Sequence
CALRdel52 Frw	ACAACTTCCTCATCACCAACG
CALRdel31 Rev	GGCCTCAGTCCAGCCCTG
CALRins5 common Frw	TAACTGCAGTGTCAGCGGTG
CALRins5 non-mutated allele Rev	TGTCCTCATCATCCTCCTTG
CALRins5 mutant Rev	TGTCCTCATCATCCTCCGAC
JAK2V617F Frw	TCCTCAGAACGTTGATGGCAG
JAK2V617F Rev	GTTTTACTTACTCTCGTCTCCACAAAA
JAK2 WT Frw	GCATTTGGTTTTAAATTATGGAGTATATG
JAK2 Rev	ATTGCTTTCCTTTTTCACAAGAT

Abbreviations: Frw: forward; Rev: reverse

Supplemental References

1. Ferreira MSV, Kirschner M, Halfmeyer I, Estrada N, Xicoy B, Isfort S, et al. Comparison of flow-FISH and MM-qPCR telomere length assessment techniques for the screening of telomeropathies. Annals of the New York Academy of Sciences. 2020;1466(1):93-103.
2. Han Y, Franzen J, Stiehl T, Gobs M, Kuo CC, Nikolic M, et al. New targeted approaches for epigenetic age predictions. BMC Biol. 2020;18(1):71.
3. Kirschner M, Maurer A, Wlodarski MW, Ventura Ferreira MS, Bouillon AS, Halfmeyer I, et al. Recurrent somatic mutations are rare in patients with cryptic dyskeratosis congenita. Leukemia. 2018;32(8):1762-1767.
4. Olschok K, Han L, de Toledo MAS, Bohnke J, Grasshoff M, Costa IG, et al. CALR frameshift mutations in MPN patient-derived iPSCs accelerate maturation of megakaryocytes. Stem cell reports. 2021;16(11):2768-2783.
5. Rufer N, Brummendorf TH, Kolvraa S, Bischoff C, Christensen K, Wadsworth L, et al. Telomere fluorescence measurements in granulocytes and T lymphocyte subsets point to a high turnover of hematopoietic stem cells and memory T cells in early childhood. The Journal of experimental medicine. 1999;190(2):157-167.
6. Rolles B, Gorgulho J, Tometten M, Roderburg C, Vieri M, Abels A, et al. Telomere Shortening in Peripheral Leukocytes Is Associated With Poor Survival in Cancer Patients Treated With Immune Checkpoint Inhibitor Therapy. Front Oncol. 2021;11:729207.
7. Krueger F, Andrews SR. Bismark: a flexible aligner and methylation caller for Bisulfite-Seq applications. Bioinformatics. 2011;27(11):1571-1572.
8. Han Y, Eipel M, Franzen J, Sakk V, Dethmers-Ausema B, Yndriago L, et al. Epigenetic age-predictor for mice based on three CpG sites. Elife. 2018;7.
9. Kalmer M, Pannen K, Lemanzyk R, Wirths C, Baumeister J, Maurer A, et al. Clonogenic assays improve determination of variant allele frequency of driver mutations in myeloproliferative neoplasms. Annals of hematology. 2022;101(12):2655-2663.
10. Baumeister J, Maie T, Chatain N, Gan L, Weinbergerova B, de Toledo MAS, et al. Early and late stage MPN patients show distinct gene expression profiles in CD34(+) cells. Annals of hematology. 2021;100(12):2943-2956.
11. Saul D, Kosinsky RL, Atkinson EJ, Doolittle ML, Zhang X, LeBrasseur NK, et al. A new gene set identifies senescent cells and predicts senescence-associated pathways across tissues. Nature communications. 2022;13(1):4827.
12. Satoh T, Toledo MAS, Boehnke J, Olschok K, Flosdorf N, Gotz K, et al. Human DC3 Antigen Presenting Dendritic Cells From Induced Pluripotent Stem Cells. Front Cell Dev Biol. 2021;9:667304.
13. Boehnke J, Atakhanov S, Toledo MAS, Schuler HM, Sontag S, Chatain N, et al. CRISPR/Cas9 mediated CXCL4 knockout in human iPS cells of polycythemia vera patient with JAK2 V617F mutation. Stem Cell Res. 2021;55:102490.

14. Cypris O, Franzen J, Frobel J, Gluck P, Kuo CC, Schmitz S, et al. Hematopoietic differentiation persists in human iPSCs defective in de novo DNA methylation. BMC Biol. 2022;20(1):141.
15. Elsafi Mabrouk MH, Goetzke R, Abagnale G, Yesilyurt B, Salz L, Cypris O, et al. The spatial self-organization within pluripotent stem cell colonies is continued in detaching aggregates. Biomaterials. 2022;282:121389.
16. Zhou W, Triche TJ, Jr., Laird PW, Shen H. SeSAMe: reducing artifactual detection of DNA methylation by Infinium BeadChips in genomic deletions. Nucleic Acids Res. 2018;46(20):e123.
17. Horvath S. DNA methylation age of human tissues and cell types. Genome Biol. 2013;14(10):R115.
18. Horvath S, Oshima J, Martin GM, Lu AT, Quach A, Cohen H, et al. Epigenetic clock for skin and blood cells applied to Hutchinson Gilford Progeria Syndrome and ex vivo studies. Aging (Albany NY). 2018;10(7):1758-1775.
19. Pidsley R, CC YW, Volta M, Lunnon K, Mill J, Schalkwyk LC. A data-driven approach to preprocessing Illumina 450K methylation array data. BMC Genomics. 2013;14:293.
20. Dagher T, Maslah N, Edmond V, Cassinat B, Vainchenker W, Giraudier S, et al. JAK2V617F myeloproliferative neoplasm eradication by a novel interferon/arsenic therapy involves PML. J Exp Med. 2021;218(2).
21. Wakita M, Takahashi A, Sano O, Loo TM, Imai Y, Narukawa M, et al. A BET family protein degrader provokes senolysis by targeting NHEJ and autophagy in senescent cells. Nature communications. 2020;11(1):1935.
22. Lomonosova E, Chinnadurai G. BH3-only proteins in apoptosis and beyond: an overview. Oncogene. 2008;27 Suppl 1(Suppl 1):S2-19.
23. Kuykendall AT, Horvat NP, Pandey G, Komrokji R, Reuther GW. Finding a Jill for JAK: Assessing Past, Present, and Future JAK Inhibitor Combination Approaches in Myelofibrosis. Cancers (Basel). 2020;12(8).
24. Ewald L, Dittmann J, Vogler M, Fulda S. Side-by-side comparison of BH3-mimetics identifies MCL-1 as a key therapeutic target in AML. Cell Death Dis. 2019;10(12):917.
25. Zhu Y, Tchkonia T, Pirtskhalava T, Gower AC, Ding H, Giorgadze N, et al. The Achilles' heel of senescent cells: from transcriptome to senolytic drugs. Aging Cell. 2015;14(4):644-658.
26. Hickson LJ, Langhi Prata LGP, Bobart SA, Evans TK, Giorgadze N, Hashmi SK, et al. Senolytics decrease senescent cells in humans: Preliminary report from a clinical trial of Dasatinib plus Quercetin in individuals with diabetic kidney disease. EBioMedicine. 2019;47:446-456.
27. Konopleva M, Martinelli G, Daver N, Papayannidis C, Wei A, Higgins B, et al. MDM2 inhibition: an important step forward in cancer therapy. Leukemia. 2020;34(11):2858-2874.
28. Lu M, Wang X, Li Y, Tripodi J, Mosoyan G, Mascarenhas J, et al. Combination treatment in vitro with Nutlin, a small-molecule antagonist of MDM2, and pegylated interferon-alpha 2a specifically targets JAK2V617F-positive polycythemia vera cells. Blood. 2012;120(15):3098-3105.
29. Liu X, Wang Y, Zhang X, Gao Z, Zhang S, Shi P, et al. Senolytic activity of piperlongumine analogues: Synthesis and biological evaluation. Bioorg Med Chem. 2018;26(14):3925-3938.
30. Hasegawa H, Yamada Y, Iha H, Tsukasaki K, Nagai K, Atogami S, et al. Activation of p53 by Nutlin-3a, an antagonist of MDM2, induces apoptosis and cellular senescence in adult T-cell leukemia cells. Leukemia. 2009;23(11):2090-2101.
31. Miller AL, Fehling SC, Garcia PL, Gamblin TL, Council LN, van Waardenburg R, et al. The BET inhibitor JQ1 attenuates double-strand break repair and sensitizes models of pancreatic ductal adenocarcinoma to PARP inhibitors. EBioMedicine. 2019;44:419-430.
32. Chen Q, Song S, Wei S, Liu B, Honjo S, Scott A, et al. ABT-263 induces apoptosis and synergizes with chemotherapy by targeting stemness pathways in esophageal cancer. Oncotarget. 2015;6(28):25883-25896.
33. Wang Y, Chang J, Liu X, Zhang X, Zhang S, Zhang X, et al. Discovery of piperlongumine as a potential novel lead for the development of senolytic agents. Aging (Albany NY). 2016;8(11):2915-2926.
34. Li Z, He S, Look AT. The MCL1-specific inhibitor S63845 acts synergistically with venetoclax/ABT-199 to induce apoptosis in T-cell acute lymphoblastic leukemia cells. Leukemia. 2019;33(1):262-266.
35. Makii C, Oda K, Ikeda Y, Sone K, Hasegawa K, Uehara Y, et al. MDM2 is a potential therapeutic target and prognostic factor for ovarian clear cell carcinomas with wild type TP53. Oncotarget. 2016;7(46):75328-75338.
36. Zoico E, Nori N, Darra E, Tebon M, Rizzatti V, Policastro G, et al. Senolytic effects of quercetin in an in vitro model of pre-adipocytes and adipocytes induced senescence. Sci Rep. 2021;11(1):23237.
37. Sahin I, Zhang S, Navaraj A, Zhou L, Dizon D, Safran H, et al. AMG-232 sensitizes high MDM2-expressing tumor cells to T-cell-mediated killing. Cell Death Discov. 2020;6:57.
38. El-Daly H, Kull M, Zimmermann S, Pantic M, Waller CF, Martens UM. Selective cytotoxicity and telomere damage in leukemia cells using the telomerase inhibitor BIBR1532. Blood. 2005;105(4):1742-1749.
39. Schafer MJ, White TA, Iijima K, Haak AJ, Ligresti G, Atkinson EJ, et al. Cellular senescence mediates fibrotic pulmonary disease. Nature communications. 2017;8:14532.
